# Smoking Behaviors and Cessation Interests Among Multiunit Subsidized Housing Tenants, Columbus, Ohio, 2011

**DOI:** 10.5888/pcd10.120302

**Published:** 2013-06-27

**Authors:** Nancy E. Hood, Amy K. Ferketich, Elizabeth G. Klein, Mary Ellen Wewers, Phyllis Pirie

**Affiliations:** Author Affiliations: Amy K. Ferketich, Elizabeth G. Klein, Mary Ellen Wewers, Phyllis Pirie, The Ohio State University College of Public Health, Columbus, Ohio.

## Abstract

**Introduction:**

Cessation services have been recommended to complement smoke-free policies in subsidized multiunit housing, but little is known about smoking- and cessation-related characteristics among subsidized housing tenants. This study examined smoking behaviors and cessation-related interests in a population of subsidized housing tenants.

**Methods:**

A face-to-face survey was conducted in August to October 2011 with a probability sample of private subsidized housing lease holders in Columbus, Ohio (N = 301, 64% response rate).

**Results:**

Almost half (47.5%) of respondents were current smokers, including smokers of cigarettes or small cigars. Smokers were less likely than nonsmokers to have health insurance and more likely to be at risk for food insecurity. Among smokers, 20.3% did not smoke daily and 35.0% smoked 5 or fewer cigarettes per day. More than half (61.3%) purchased single cigarettes in the past month, with higher rates among nondaily smokers. Most smokers intended to quit within 6 months or less (60.1%) and were interested in using nicotine replacement therapy (NRT) (65.0%). Most respondents had Medicaid but only 30.4% knew Medicaid covered cessation medications.

**Conclusions:**

This population of subsidized housing tenants had high rates of smoking, including light smoking. Interest in NRT was high and access can be improved by increasing awareness of Medicaid coverage among clients and health care providers. However, more research is needed about scalable, evidence-based cessation strategies for low-socioeconomic status and light smokers. Strategies to address environmental factors such as availability of single cigarettes should also be considered in parallel with smoke-free policies.

## MEDSCAPE CME

Medscape, LLC is pleased to provide online continuing medical education (CME) for this journal article, allowing clinicians the opportunity to earn CME credit.

This activity has been planned and implemented in accordance with the Essential Areas and policies of the Accreditation Council for Continuing Medical Education through the joint sponsorship of Medscape, LLC and Preventing Chronic Disease. Medscape, LLC is accredited by the ACCME to provide continuing medical education for physicians.

Medscape, LLC designates this Journal-based CME activity for a maximum of 1 **AMA PRA Category 1 Credit(s)™**. Physicians should claim only the credit commensurate with the extent of their participation in the activity.

All other clinicians completing this activity will be issued a certificate of participation. To participate in this journal CME activity: (1) review the learning objectives and author disclosures; (2) study the education content; (3) take the post-test with a 70% minimum passing score and complete the evaluation at www.medscape.org/journal/pcd (4) view/print certificate.

**Release date: June 26, 2013; Expiration date: June 26, 2014**

### Learning Objectives

Upon completion of this activity, participants will be able to:

- Analyze smoking patterns and outcomes among persons of low socioeconomic status
- Evaluate attitudes and knowledge regarding smoking cessation among subsidized housing tenants
- Distinguish the preferred smoking cessation aid among subsidized housing tenants

**EDITORS**

Caran Wilbanks, Editor, *Preventing Chronic Disease*. Disclosure: Caran Wilbanks has disclosed the following relevant financial relationship: Partner is employed by McKesson Corporation.

**CME AUTHOR**

Charles P. Vega, MD, Associate Professor and Residency Director, Department of Family Medicine, University of California, Irvine. Disclosure: Charles P. Vega, MD, has disclosed no relevant financial relationships.

**AUTHORS AND CREDENTIALS**

Disclosures: Nancy E. Hood, PhD, MPH; Amy K. Ferketich, PhD; Elizabeth G. Klein, PhD, MPH; Mary Ellen Wewers, PhD, MPH; Phyllis Pirie, PhD, have disclosed no relevant financial relationships.

Affiliations: Amy K. Ferketich, Elizabeth G. Klein, Mary Ellen Wewers, Phyllis Pirie, The Ohio State University College of Public Health, Columbus, Ohio.

## Introduction

Smoke-free policies in publicly subsidized multiunit housing (MUH) are increasingly being promoted to protect nonsmokers of low socioeconomic status (SES) from involuntary secondhand smoke exposure in the home (1). Although these policies do not require smokers to quit smoking, it is recommended that cessation services accompany such policies (1). In the only outcome evaluation of a smoke-free policy in subsidized MUH, 15% of smokers reported quitting within about 18 months after policy implementation (2). Although self-reported data were not biochemically validated, this finding at least suggests smokers may be interested in quitting in response to smoke-free policies.

Smoking behaviors and cessation interests among smokers in subsidized housing are not well understood, and such information could be used to inform cessation programs. Estimates of smoking rates among subsidized housing tenants suggest that they exceed national estimates for the lowest SES groups (3,4). In general, low-SES smokers have higher levels of nicotine dependence and are less likely to intend to quit or to successfully quit smoking compared with their higher-SES counterparts (5,6). However, mean annual household income for subsidized housing tenants is approximately $12,000 (7), whereas most definitions of low SES in previous studies include notably higher incomes. Cigarette consumption could be lower among subsidized housing tenants because of limited financial resources or higher because of financial and other stressors. Tenants may be less interested in quitting because of the high prevalence of smoking or more interested because of high financial stress (8).

Evidence-based treatments such as behavioral counseling and nicotine replacement therapy (NRT) double a smoker’s chance of quitting successfully and are recommended for all smokers, including those who are low SES (9). However, low-SES smokers are less likely to use NRT or other cessation aids compared with their higher-SES counterparts (10). Barriers to use include lack of knowledge about the effectiveness of such aids and limited availability (11). Although low-SES smokers who visit health care providers are as likely as other smokers to receive advice about quitting from health care providers (10), they are less likely to receive assistance with quitting (12).

Our study examined smoking behaviors and cessation-related interests among smokers in subsidized MUH collected as part of a larger survey about voluntary home smoking restrictions and support for smoke-free housing policies. There were 3 primary aims: 1) compare sociodemographic and health characteristics between smokers and nonsmokers; 2) describe smoking behaviors and cessation-related interests among smokers; and 3) identify differences in sociodemographic, smoking, and cessation-related characteristics between smokers with different levels of cigarette consumption. The third aim was identified based on preliminary analyses that showed that the survey population had a large proportion of nondaily and light smokers.

## Methods

### Sample

The study population was tenants in approximately 1,000 subsidized MUH units in 184 buildings across 5 urban neighborhoods and managed by a private company in Columbus, Ohio. No buildings or units were covered by a smoke-free housing policy. By using units that were occupied as of July 2011 (n = 914), a stratified random sample (n = 475) was selected, and the primary lease holder in each unit was eligible to participate in the study. Administrative data provided by the property management company were used to stratify units by the age of the youngest child (<5 years; 5–17 years; or no children <18 years) because this factor was expected to be associated with voluntary home smoking restrictions and support for smoke-free policies, which were the primary outcomes in the larger study. Units confirmed as vacant by the property management company at the time they were fielded were ineligible (n = 3).

### Data collection

An interviewer-administered, face-to-face survey was conducted at tenants’ homes from August to October 2011. A personalized letter was sent to lease holders in selected units 1 week before the first in-person visit. Teams of 2 interviewers (1 community resident and 1 graduate student) made at least 5 in-person attempts to contact each lease holder at different days and times. Visits took an average of 27.0 minutes to complete and participants were given a $5 grocery store gift card. The study was approved by the university’s institutional review board, and participants provided informed consent.

### Measures

All measures were self-reported. Sociodemographic characteristics collected were age, race/ethnicity, sex, age of youngest child under 18 years living in household, presence of a child with asthma diagnosis, educational attainment, and employment status. Health-related characteristics collected were health insurance status, type of insurance (Medicaid, Medicare, or private), general health status, and physical limitations during the past month (not at all or very little vs somewhat or quite a lot). Food insecurity was measured by using a previously validated 2-item scale (13); those who reported at least 1 of the following statements was true often or sometimes in the past 12 months were classified as at risk for food insecurity: “We worried whether our food would run out before we got money to buy more,” and “The food we bought just didn’t last and we didn’t have money to get more.”

All participants were asked if they had smoked at least 100 cigarettes in their lifetime and how many days per week they smoke cigarettes now. Interviewers instructed respondents to include small cigars in response to these questions because there was not a separate question about small cigar use. Never smokers had never smoked 100 cigarettes and smoked zero days per week now. Former smokers had smoked 100 cigarettes in their lifetime but smoked zero days now. Current smokers smoked 1 to 7 days per week now regardless of whether they had smoked 100 cigarettes in their lifetime; this definition is appropriate for measuring lower levels of cigarette consumption that are common among young adult and nonwhite smokers (14).

Current smokers also reported how many cigarettes they usually smoke on days they smoke. Cigarette consumption levels were classified as nondaily (1-6 days/week), light daily (1–5 cigarettes per day [CPD]), 7 d/wk), and nonlight daily (≥6 CPD, 7 d/wk). Categorical responses for time to first cigarette after waking (TTF) were dichotomized into 30 minutes or less and more than 30 minutes. Strength of urges to smoke in the past 24 hours (15) was dichotomized as none, slight, or moderate versus strong or very strong. Current smokers were asked, “Is your usual cigarette brand menthol or nonmenthol?” and “How often in the past month have you bought cigarettes loose out of the pack?” (16).

Based on the 2-question stage of change measure (17), smokers who intended to quit within 6 months or 30 days were grouped versus those who did not intend to quit. Although intending to quit within 30 days is a better predictor of short-term cessation than within 6 months, both predict future quit attempts (17), and there were too few respondents who intended to quit within 30 days to conduct separate analyses for this group. Current smokers were also asked, “If you decided to quit smoking, how interested would you be in using each of the following to help you quit if they were free?” Methods were nicotine patches, gum, or lozenges; other medication like Chantix; face-to-face counseling program; telephone counseling program (also called a quitline); and Internet counseling program. Response options included not at all, very, or somewhat interested. Respondents with health insurance were asked whether their insurance would pay for medications, face-to-face programs, or telephone programs to help people quit smoking.

### Data analysis

Because our study focused on current smokers, we grouped never and former smokers as nonsmokers. We compared sociodemographic and health characteristics between current and nonsmokers and across cigarette consumption levels. Chi-square tests of association with Rao-Scott adjustment for the stratified survey design were used for categorical variables and Kruskal–Wallis tests for continuous variables. Survey logistic models with adjustment for stratification were used for most multivariable analyses. Exact logistic regression models that also controlled for survey design strata were used for analyses comparing smoking-related behaviors and characteristics across consumption levels due to zero or small sample sizes. Analyses related to awareness of insurance benefits were limited to respondents who reported Medicaid coverage because cessation-related benefits are not consistent across other insurance types. Analyses were conducted with SAS 9.2 (SAS Institute Inc, Cary, North Carolina).

## Results

Completed surveys were obtained from lease holders in 301 units (63.8% response rate). Analysis of administrative data from the property management company showed that nonrespondents did not differ from respondents in lease holder age, age of youngest child, or neighborhood of residence.

### Characteristics of smokers and nonsmokers

Overall, 47.5% (n = 143) of respondents were current smokers, 12.3% (n = 37) were former smokers, and 40.2% were never smokers (n = 121); therefore, 52.5% were nonsmokers. Fifteen current smokers had not smoked 100 cigarettes in their lifetime. Smokers were less likely than nonsmokers to have at least a high school education, be employed, or have health insurance, and were more likely to have physical limitations or be at risk of food insecurity (Table 1). After controlling for these differences, smokers were less likely to have health insurance (adjusted odds ratio [AOR], 0.45; 95% confidence interval [CI], 0.21–1.00) and more likely to be at risk of food insecurity (AOR, 1.73; 95% CI, 1.07–2.81).

**Table 1:** Sociodemographic and Health Characteristics of Tenants in Private, Multiunit Subsidized Housing, by Smoking Status, Columbus, Ohio, 2011

Variable	Total (N = 301)	Nonsmokers^a^ (n = 158)	Smokers (n = 143)	*P* Value^b^

	No. (%^c^)			
**Demographic characteristics**				
**Age, y, median**	24.8	23.9	27.4	.23
**Female**	260 (86.4)	140 (88.6)	120 (83.9)	.24
**African American**	251 (83.7)	129 (81.6)	122 (85.9)	.32
**Age of youngest child**				.43
No children	82 (27.2)	41 (25.9)	41 (28.7)	
<5 years	167 (55.5)	93 (58.9)	74 (51.7)	
5–17 years	52 (17.3)	24 (15.2)	28 (19.6)	
**Has child with asthma**	70 (23.3)	36 (22.8)	34 (23.8)	.84
**Less than high school graduate**	88 (29.2)	36 (22.8)	52 (36.4)	.01
**Employed full- or part-time**	100 (33.2)	64 (40.5)	36 (25.2)	.005
**Health-related characteristics**				
Has health insurance	265 (88.3)	145 (92.4)	120 (83.9)	.02
Fair or poor general health status	99 (32.9)	44 (27.8)	55 (38.5)	.05
Had physical health limitations in past month	91 (30.2)	45 (28.5)	46 (32.2)	.49
At risk for food insecurity^d^	149 (49.7)	65 (41.4)	84 (58.7)	.003

a Includes never and former smokers.

b Statistical tests were χ^2^ tests of association (with Rao-Scott adjustment for survey design) for categorical variables and Kruskal-Wallis tests for the continuous variable (age).

c Denominators for percentages are not always the total n for the group because of missing data.

d Respondents who reported that at least 1of 2 statements about food insecurity were true often or sometimes in the past 12 months (13) were classified as at risk for food insecurity.

### Smoking-related behaviors

Among smokers, 20.3% were nondaily, 35.0% were light daily, and the remaining 44.8% were nonlight daily. Only 14.7% of all smokers consumed more than 10 CPD. Median age was the only sociodemographic or health characteristic that differed significantly by consumption level (*P* = .002); age was lower for nondaily (23.2 years) and light daily (25.0 years) than for nonlight daily smokers (31.5 years). After controlling for age — the only demographic variable that differed by consumption level — the odds of smoking within 30 minutes of waking and having strong urges to smoke remained significantly higher for nonlight daily versus light daily smokers (AOR, 4.5; 95% CI, 1.8–11.7, and AOR, 4.2; 95% CI, 1.6–12.3, respectively). The odds of smoking within 30 minutes of waking were lower for nondaily smokers compared with light daily smokers (AOR, 0.1; 95% CI, 0.0–0.5). Odds of strong urges are not reported for nondaily smokers because of zero cell size. More than half of smokers had ever purchased single cigarettes in the past month (Table 2), including 27.5% who purchased singles frequently (ie, many times or just about all the time). Nondaily smokers were more likely to have purchased singles in the past month compared with nonlight daily smokers after controlling for age (AOR, 5.6; 95% CI, 1.6–25.6).

**Table 2:** Smoking-Related Behaviors and Characteristics of Smokers in Private, Multiunit Subsidized Housing, By Cigarette Consumption Level, Columbus, Ohio, 2011

Behavior or Characteristic	Total (n = 143)	Nondaily^a^ (n = 29)	Light daily^a^ (n = 50)	Nonlight daily^a^ (n = 64)	*P* Value^b^

	No. (%^c^)				
**TTF ≤30 min^d^**	59 (41.5)	1 (3.6)	16 (32.0)	42 (65.6)	<.001
**Very strong/strong urges to smoke^d^**	39 (27.3)	0	8 (16.0)	31 (48.4)	<.001
**Usual brand is menthol^e^**	111 (90.2)	16 (84.2)	39 (95.1)	56 (88.9)	.36
**Bought singles in past month^f^**	87 (61.3)	25 (86.2)	33 (66.0)	29 (46.0)	<.001
**Intend to quit within 6 months**	86 (60.1)	19 (65.5)	32 (64.0)	35 (54.7)	.49
**Interested in using cessation aids^g^**					
Nicotine replacement therapy	91 (65.0)	14 (48.3)	32 (64.0)	45 (73.8)	.06
Other medications	52 (36.9)	8 (27.6)	15 (30.0)	29 (46.8)	.10
Face-to-face counseling	56 (39.7)	9 (31.0)	18 (36.0)	29 (46.8)	.29
Telephone counseling	53 (37.6)	10 (34.5)	17 (34.0)	26 (41.9)	.64
Internet counseling	31 (22.0)	5 (17.2)	10 (20.0)	16 (25.8)	.60

Abbreviation: TTF, time to first cigarette after waking.

a Nondaily = smoke on 1 to 6 d/wk; light daily = 1 to 5 cigarettes per day, 7 days per week; nonlight daily = 6 or more cigarettes per day, 7 days per week.

b Statistical tests were χ^2^ tests of association (with Rao-Scott adjustment for survey design).

c Denominators for percentages are not always the total n for the group because of missing data.

d Nondaily smokers were excluded from comparisons across consumption levels because of small or zero cell size.

e “Don’t know” (n = 19) and refused (n = 1) responses were excluded; “don’t know” responses were primarily from respondents who only smoked small cigars.

f Defined as “ever” (ie, a few times, many times, or just about all of the time) bought single cigarettes loose out of the pack in the past month (versus “never”).

g “Interested” was defined as “very” or “somewhat” versus “not at all.”

### Cessation intentions and interests

More than half of smokers intended to quit within the next 6 months or less (Table 2), including 25.9% within the next month and an additional 34.3% within 6 months. Intentions did not differ significantly by consumption level. Smokers were most interested in using NRT as a cessation aid if they decided to quit smoking (Table 2). Interest in using cessation aids increased with consumption levels but differences were not significant (Table 2).

### Awareness of Medicaid coverage

More than three-fourths (78.7%) of respondents reported having Medicaid insurance, which covers all cessation medications but no counseling (face-to-face or telephone) in Ohio. At the time of the survey, low-income smokers could receive some telephone counseling through the Ohio Tobacco Quitline supported by the state. Of respondents with Medicaid, less than one-third (30.4%) stated that Medicaid would pay for cessation medications. Lower percentages reported that Medicaid would pay for face-to-face (19.4%) or telephone (17.3%) counseling. There were no differences in knowledge of medication coverage between smokers and nonsmokers or by quit intentions, consumption level, or interest in using NRT or other medications among smokers.

## Discussion

A better understanding of smoking behaviors and cessation interests among low-SES smokers in subsidized MUH will help inform cessation and other interventions to accompany smoke-free housing policies. Strengths of this study included a probability sample and high response rate. Because they qualified for subsidized housing, all respondents had very low incomes; additionally, they were predominantly African American and female. Our findings were similar to national estimates for African Americans (18) in that almost one-fourth of smokers did not smoke every day. However, light daily smoking was 3 times more common than among African American smokers in general (18). Light smoking is a concern because it causes similar risks of cardiovascular disease as heavier smoking and higher risks of lung and other cancers, respiratory disease, and all-cause mortality than nonsmoking (19).

Smokers reported notably higher rates of quit intentions than other low-SES populations (5), possibly because of high rates of financial stress (almost 60% reported food insecurity in the past year) (8) or nondaily and light daily smoking (20). Although intentions to quit have not been associated with long-term cessation, they are associated with short-term cessation among smokers with previous quit attempts and with increased quit attempts (17). Intentions to quit may also reflect receptivity to smoking cessation supports (17). However, because both low-SES and African American smokers have lower cessation rates than other smokers (5,10), interventions are needed to transform high intentions into successful cessation.

Few studies have examined the effectiveness of evidence-based cessation treatment among very low-SES, light, or nondaily smokers. Guidelines recommend evidence-based cessation aids for low-SES smokers (9) but some cessation aids have been less effective among low-SES (21,22) and light (23) smokers. A community-based intervention with women living in public housing that included nicotine patches and counseling was effective at 6 months (24). However, only daily smokers were eligible for the study and the intervention would likely be cost-prohibitive to implement across a large number of subsidized housing settings. More research is needed to design scalable, culturally appropriate strategies to address a range of consumption levels and contextual realities (eg, food insecurity, availability of single cigarettes) of very low-SES smokers.

The sale of single cigarettes is illegal, and observational data from tobacco retail outlets do not typically reveal single cigarette sales (25). However, the high rate of self-reported single cigarette purchasing was consistent with a previous study of young urban African American adults in Baltimore (16). Smokers purchase single cigarettes for different reasons, including convenience, low immediate costs, attempts to quit or cut back, and urges to smoke when they see singles for sale (16,26). More research is needed to determine whether single cigarettes serve to maintain or treat dependence among very low-SES populations, and whether singles are primarily purchased from commercial venues or other individuals.

For subsidized housing tenants who have high levels of nicotine dependence, Medicaid ensures access to cessation medications. Although awareness rates were similar to other surveys of smokers who have Medicaid insurance (11,27,28), 70% of respondents were not aware of this coverage; we found no differences by smoking status, intentions to quit, or intentions to use cessation medications. Several previous studies have evaluated interventions to increase awareness and use of insurance coverage for treatment of tobacco dependence. The most promising was a health maintenance organization–based communication campaign that increased use of cessation medications and the telephone quitline among Medicaid smokers in Wisconsin compared with fee-for-service Medicaid enrollees without a campaign (29). The success of this intervention highlights the importance of involving health care providers, many of whom are also unaware of Medicaid cessation benefits (28), and using integrated, multicomponent strategies. Campaigns to increase awareness and use of Medicaid cessation coverage among MUH tenants could focus on settings where most tenants receive health care services as well as housing offices, tenant newsletters or mailings, and resident support services. Additional barriers, such as perceived effectiveness of medications, must be addressed in parallel with awareness of benefits (11).

### Limitations

Smokers may have been less likely to respond than nonsmokers, and self-reported smoking status was not biochemically verified. However, this means that the reported smoking rate, which was much higher than in the general population, is conservative. Sample sizes were small for nondaily smokers, which limited power for comparisons across consumption levels. Generalizability to all subsidized housing populations may be limited. In particular, age was associated with consumption levels, and older tenants were underrepresented in the study population compared with national estimates for subsidized housing tenants. However, a large minority of all subsidized housing tenants nationally are African American and have children, and a majority are female (7), making them comparable to our study population.

### Conclusions

Smoking in general, and light smoking specifically, was much more common in this subsidized housing population compared with the general population. Medicaid coverage for cessation medications should be promoted to tenants and health care providers because most daily smokers were interested in using NRT but most did not know Medicaid covers these medications. Policies and interventions to address environmental factors such as availability of single cigarettes should be considered in parallel with smoke-free housing policies, especially because few tobacco dependence treatments have been tested or shown to be effective with very low-SES or light smokers.

## Figures and Tables

**Table:** 

**1. The activity supported the learning objectives.**				
**Strongly Disagree**				**Strongly Agree**
1	2	3	4	5
**2. The material was organized clearly for learning to occur.**				
**Strongly Disagree**				**Strongly Agree**
1	2	3	4	5
**3. The content learned from this activity will impact my practice.**				
**Strongly Disagree**				**Strongly Agree**
1	2	3	4	5
**4. The activity was presented objectively and free of commercial bias.**				
**Strongly Disagree**				**Strongly Agree**
1	2	3	4	5

## Post-Test Information

To obtain credit, you should first read the journal article. After reading the article, you should be able to answer the following, related, multiple-choice questions. To complete the questions (with a minimum 70% passing score) and earn continuing medical education (CME) credit, please go to http://www.medscape.org/journal/pcd. Credit cannot be obtained for tests completed on paper, although you may use the worksheet below to keep a record of your answers. You must be a registered user on Medscape.org. If you are not registered on Medscape.org, please click on the "Register" link on the right hand side of the website to register. Only one answer is correct for each question. Once you successfully answer all post-test questions you will be able to view and/or print your certificate. For questions regarding the content of this activity, contact the accredited provider, CME@medscape.net. For technical assistance, contact CME@webmd.net. American Medical Association's Physician's Recognition Award (AMA PRA) credits are accepted in the US as evidence of participation in CME activities. For further information on this award, please refer to http://www.ama-assn.org/ama/pub/about-ama/awards/ama-physicians-recognition-award.page. The AMA has determined that physicians not licensed in the US who participate in this CME activity are eligible for **AMA PRA Category 1 Credits™**. Through agreements that the AMA has made with agencies in some countries, AMA PRA credit may be acceptable as evidence of participation in CME activities. If you are not licensed in the US, please complete the questions online, print the AMA PRA CME credit certificate and present it to your national medical association for review.

## Post-Test Questions

### Article Title: Smoking Behaviors and Cessation Interests Among Multiunit Subsidized Housing Tenants, Columbus, Ohio, 2011

**CME Questions**

1. You are seeing a 25-year-old African American woman for a 2-week history of cough. In taking her social history, you find out that she lives in subsidized housing and that she currently smokes several cigarettes per day. What should you consider regarding characteristics of smokers in the current study and light smokers overall?
  - 85% of all participants in the current study were current smokers
  - Smoking was associated with higher rates of food insecurity in the current study
  - Light smoking does not significantly increase the risks of cancer or all-cause mortality compared with never smoking
  - Smokers were more likely to have health insurance in the current study
2. Which of the following statements regarding smoking patterns among participants in the current study is most accurate?
  - A minority of smokers consumed more than 10 cigarettes per day
  - Light daily smokers were more likely to smoke within 30 minutes of waking compared with nonlight daily smokers
  - There was no difference in the urge to smoke in comparing light daily smokers with nonlight daily smokers
  - The purchase of single cigarettes was very rare, as such sales are illegal
3. You discuss the possibility of smoking cessation with the patient. What should you consider regarding patient knowledge and attitudes regarding smoking cessation in the current study?
  - Less than 10% of smokers intended to quit in the next 6 months
  - The most important factor in influencing the intent to quit smoking was heavier cigarette consumption
  - Only 30% of participants knew that Medicaid would pay for medications to help quit smoking
  - Participants who intended to quit smoking were much more aware of Medicaid coverage compared with those who had no intention to quit
4. Which of the following options was the most favored smoking cessation aid among participants in the current study?
  - Telephone counseling
  - Face-to-face counseling
  - Nicotine replacement therapy
  - Oral medications

**Evaluation**
